# Ribosome-Associated Mba1 Escorts Cox2 from Insertion Machinery to Maturing Assembly Intermediates

**DOI:** 10.1128/MCB.00361-16

**Published:** 2016-10-28

**Authors:** Isotta Lorenzi, Silke Oeljeklaus, Christin Ronsör, Bettina Bareth, Bettina Warscheid, Peter Rehling, Sven Dennerlein

**Affiliations:** a Department of Cellular Biochemistry, University Medical Center Göttingen, GZMB, Göttingen, Germany; b Faculty of Biology, Department of Biochemistry and Functional Proteomics, University of Freiburg, Freiburg, Germany; c BIOSS Center for Biological Signalling Studies, University of Freiburg, Freiburg, Germany; d Max Planck Institute for Biophysical Chemistry, Göttingen, Germany

## Abstract

The three conserved core subunits of the cytochrome *c* oxidase are encoded by mitochondria in close to all eukaryotes. The Cox2 subunit spans the inner membrane twice, exposing the N and C termini to the intermembrane space. For this, the N terminus is exported cotranslationally by Oxa1 and subsequently undergoes proteolytic maturation in Saccharomyces cerevisiae. Little is known about the translocation of the C terminus, but Cox18 has been identified to be a critical protein in this process. Here we find that the scaffold protein Cox20, which promotes processing of Cox2, is in complex with the ribosome receptor Mba1 and translating mitochondrial ribosomes in a Cox2-dependent manner. The Mba1-Cox20 complex accumulates when export of the C terminus of Cox2 is blocked by the loss of the Cox18 protein. While Cox20 engages with Cox18, Mba1 is no longer present at this stage. Our analyses indicate that Cox20 associates with nascent Cox2 and Mba1 to promote Cox2 maturation cotranslationally. We suggest that Mba1 stabilizes the Cox20-ribosome complex and supports the handover of Cox2 to the Cox18 tail export machinery.

## INTRODUCTION

The mitochondrial oxidative phosphorylation system generates the bulk of cellular ATP. During this process, electrons are transferred from NADH and FADH_2_ to the mitochondrial respiratory chain, resulting in the generation of a proton gradient across the inner mitochondrial membrane that drives ATP synthesis by the F_1_F_o_ ATP synthase. Four multisubunit protein complexes constitute the respiratory chain. The terminal electron transfer enzyme is cytochrome *c* oxidase, which reduces molecular oxygen to water. The enzymatic activity of the oxidase resides in the highly conserved central subunits Cox1 and Cox2, which coordinate the heme and copper cofactors responsible for electron transport (1–5). Twelve polypeptides of dual genetic origin build the cytochrome *c* oxidase of the yeast Saccharomyces cerevisiae. The majority of these subunits are encoded by the nucleus and need to be imported into the organelle. In addition, the enzyme contains three core subunits encoded by the mitochondria, Cox1, Cox2, and Cox3, which are cotranslationally inserted into the inner mitochondrial membrane by the coordinated action of mitochondrial ribosomes and the protein export machinery (6, 7).

After membrane insertion of the core subunits, assembly of the enzyme complex is initiated on Cox1. Concomitantly, specific factors, so-called assembly factors, assist with the maturation and incorporation of cytochrome *c* oxidase subunits into the complex. The current concept of the assembly process proposes a stepwise process through a series of assembly intermediates (8–10).

However, how the core subunits encoded by the mitochondria are handed over from the export machinery to the assembly intermediates is not understood.

Studies in the yeast Saccharomyces cerevisiae have led to the identification of several components involved in the biogenesis of the Cox2 core subunit. Cox2 is embedded in the inner membrane with two transmembrane spans and exposes its N- and C-terminal domains to the intermembrane space. In S. cerevisiae and in plants, Cox2 is expressed with a cleavable N-terminal presequence of 12 to 15 amino acids (11, 12). Prior to presequence cleavage, Cox2 targeting of the membrane begins with the action of the membrane-associated translational activator Pet111, which recognizes the 5′ untranslated region of *COX2* mRNA (13–16). Cotranslational insertion of the Cox2 N-terminal tail together with the first transmembrane span depends on the export machinery, Oxa1 (17–19). The peripheral inner membrane protein Mba1 cooperates with Oxa1 in the insertion of mitochondrial translation products (20–25). Mba1 is thought to align the ribosomal exit tunnel with the export machinery (23). Once it is exported into the intermembrane space, the Cox2 N terminus is processed by the Imp1/Imp2/Som1 protease complex (26–28) in a reaction requiring the Cox2-specific chaperone, Cox20 (12, 29). Cox20 and its human homolog (COX20) comprise two transmembrane spans exposing the N and C termini into the intermembrane space (30, 31). In addition, Cox20 participates in the C-terminal export of Cox2 (12). Cox18, which is dedicated to Cox2 C-terminal translocation, associates with Cox20 in a Cox2-dependent manner, suggesting a possible role for Cox20 in handing over the processed Cox2 to Cox18 (32–35). However, Cox20 is also present in organisms that lack a Cox2 presequence. This fact is indicative of an additional function for Cox20, aside from Cox2 processing. In humans, a mutation in COX20 has been linked to muscle hypotonia and ataxia with cytochrome *c* oxidase deficiency (31, 36).

Here, we carried out a comprehensive analysis of the Cox20 interaction network. The characterization of Cox20-containing complexes defined Cox20 to be a scaffold protein that interacts with the mitochondrial ribosome, linking its function to Cox2 translation. Interestingly, we demonstrate that the ribosome-binding protein Mba1 associates with Cox20 in a defined complex in a Cox2-dependent manner. In addition, our studies show that Mba1 associates with the ribosome and Cox2 assemblies in a dynamic manner. Based on these findings, we propose that Mba1 escorts newly synthesized Cox2 from the insertion machinery to maturing assembly intermediates in a Cox20-dependent manner.

## MATERIALS AND METHODS

### Yeast strains and growth conditions.

The S. cerevisiae strains used in this study are listed in Table 1. All strains with the exception of the *imp1*Δ strain are congenic to strain YPH499; the *imp1*Δ strain is derived from BY4741 (37, 38). Deletions of *MBA1*, *PET111*, *COX18*, *IMP1*, and the sequence corresponding to the N terminus of *COX20* were generated by introduction of *HIS3MX6*, *klTRP1*, and *natNT2* cassettes. Protein A (ProtA)-tagged versions of *COX20*, *MBA1*, and *COX18* were created by homologous recombination using PCR-derived cassettes amplified from plasmids pYM10 and pYM9 (39).Yeast strains were grown on nonfermentable medium, 1% yeast extract, 2% peptone, 3% glycerol (YPG), or fermentable medium, 1% yeast extract, 2% peptone, 2% glucose (YPD) or 1% yeast extract, 2% peptone, 2% galactose (YPGal). Unless otherwise indicated, yeast cells were grown at 30°C with shaking. Yeast growth tests were performed by adjusting precultures to an optical density at 600 nm of 0.3, spotting serial 1:10 dilutions onto YPD and YPG agar plates, and incubating the yeast cells for 3 days at the temperatures indicated below. Mitochondria were isolated from yeast cells grown on YPG or YPGal medium at 30°C as previously described (40). Mitochondrial translation inhibition treatment was performed as follows: yeast cells were grown until mid-log-phase and incubated with 6 mM chloramphenicol for 3 h before mitochondrial isolation (41).

**TABLE 1:** Yeast strains used in this study

Strain	Genotype	Authors (reference) or source
YPH499	*MAT***a** *ade2*-*101 his3*-Δ*200 leu2*-Δ*1 ura3*-*52 trp1*-Δ*63 lys2*-*801*	Sikorski and Hieter (38)
YPH499 for SILAC	*MAT***a** *ade2*-*101 his3*-Δ*200 leu2*-Δ*1 ura3*-*52 trp1*-Δ*63 lys2*-*801 arg4*::*kanMX4*	Alkhaja et al. (44)
Cox20^ProtA^ (ILY59)	*MAT***a** *ade2*-*101 his3*-Δ*200 leu2*-Δ*1 ura3*-*52 trp1*-Δ*63 lys2*-*801 cox20*::*cox20*-TEV-ProtA-7His-*HIS3MX6*	This study
Cox20^ProtA^ for SILAC (ILY119)	*MAT***a** *ade2*-*101 his3*-Δ*200 leu2*-Δ*1 ura3*-*52 trp1*-Δ*63 lys2*-*801 arg4*::*kanMX4 cox20*::*cox20*-TEV-ProtA-7His-*HIS3MX6*	This study
*mba1*ΔCox20^ProtA^ (ILY110)	*MAT***a** *ade2*-*101 his3*-Δ*200 leu2*-Δ*1 ura3*-*52 trp1*-Δ*63 lys2*-*801 mba1*::*HIS3MX6 cox20*::*cox20*-TEV-ProtA-7His-*kanMX4*	This study
*pet111*ΔCox20^ProtA^ (ILY135)	*MAT***a** *ade2*-*101 his3*-Δ*200 leu2*-Δ*1 ura3*-*52 trp1*-Δ*63 lys2*-*801 pet111*::*klTRP1 cox20*::*cox20*-TEV-ProtA-7His-*HIS3MX6*	This study
*imp1*ΔCox20^ProtA^ (ILY133)	*MAT***a** *ade2*-*101 his3*-Δ*200 leu2*-Δ*1 ura3*-*52 trp1*-Δ*63 lys2*-*801 imp1*::*klTRP1 cox20*::*cox20*-TEV-ProtA-7His-*HIS3MX6*	This study
*cox20*Δ (ILY60)	*MAT***a** *ade2*-*101 his3*-Δ*200 leu2*-Δ*1 ura3*-*52 trp1*-Δ*63 lys2*-*801 cox20*Δ*1*-*81*::*natNT2*	This study
Mba1^ProtA^ (ILY93)	*MAT***a** *ade2*-*101 his3*-Δ*200 leu2*-Δ*1 ura3*-*52 trp1*-Δ*63 lys2*-*801 mba1*::*mba1*-TEV-ProtA-7His-*HIS3MX6*	This study
*cox20*ΔMba1^ProtA^ (ILY104)	*MAT***a** *ade2*-*101 his3*-Δ*200 leu2*-Δ*1 ura3*-*52 trp1*-Δ*63 lys2*-*801 cox20*Δ*1*-*81*::*natNT2 mba1*::*mba1*-TEV-ProtA-7His-*HIS3MX6*	This study
*pet111*ΔMba1^ProtA^ (ILY134)	*MAT***a** *ade2*-*101 his3*-Δ*200 leu2*-Δ*1 ura3*-*52 trp1*-Δ*63 lys2*-*801 pet111*::*klTRP1 mba1*::*mba1*-TEV-ProtA-7His-*HIS3MX6*	This study
*cox18*ΔMba1^ProtA^ (ILY153)	*MAT***a** *ade2*-*101 his3*-Δ*200 leu2*-Δ*1 ura3*-*52 trp1*-Δ*63 lys2*-*801 cox18*::*klTRP1 mba1*::*mba1*-TEV-ProtA-7His-*HIS3MX6*	This study
Cox18^ProtA^ (ILY94)	*MAT***a** *ade2*-*101 his3*-Δ*200 leu2*-Δ*1 ura3*-*52 trp1*-Δ*63 lys2*-*801 cox18*::*cox18*-TEV-ProtA-7His-*HIS3MX6*	This study
*imp1*Δ	*MAT***a** *his3*Δ*1 leu2*Δ*0 met15*Δ*0 ura3*Δ*0 imp1*::*kanMX4*	Euroscarf

### *In organello* labeling of mitochondrial translation products.

Mitochondrial translation products were radiolabeled for 20 min with 20 μM [^35^S]methionine (10 mCi/ml) at 30°C, as described previously (42). Labeling reactions were stopped by addition of excess methionine (15 mM). Prior to IgG chromatography, mitochondria were reisolated and washed with SEM buffer (250 mM saccharose, 1 mM EDTA, 10 mM MOPS [morpholinepropanesulfonic acid]). To detect radioactively labeled proteins, storage phosphor screens (GE Healthcare) were used, and signals were digitized using a scanner (Storm820; GE Healthcare).

### IgG affinity chromatography.

CNBr-activated Sepharose (GE Healthcare) was coupled to human IgG (Sigma-Aldrich) according to the producer's specifications. Protein complexes were purified from mitochondria isolated from strains expressing the ProtA-tagged proteins of interest. For this purpose, mitochondria were solubilized in 1% digitonin buffer containing 150 mM NaCl, 50 mM Tris-HCl, pH 7.5, 10% glycerol, and 2 mM phenylmethylsulfonyl fluoride (PMSF) for 30 min on ice. Solubilized material was clarified by centrifugation at 20,000 × *g* and 4°C for 10 min and added to IgG-Sepharose beads at a ratio of 10 μl beads per 1 mg protein. Binding was carried out for a period of 2 h at 4°C with mild agitation. The beads were then washed 10 times with washing buffer containing 0.3% digitonin. Bound proteins were eluted with 0.1 M glycine, pH 2.8, or cleaved overnight at 4°C with 0.4 mg/ml acetylated tobacco etch virus (AcTEV; Thermo Fisher Scientific) protease. Eluates were mixed with loading dye and subsequently analyzed by SDS-PAGE, blue native (BN)-PAGE, or mass spectrometry (MS).

### Miscellaneous.

Standard methods were used for SDS-PAGE and Western blotting. Detection of primary antibody-protein complexes was performed using horseradish peroxidase-coupled secondary antibodies (Jackson ImmunoResearch). Signals were detected using an enhanced chemiluminescence system (Thermo Scientific) and exposed on X-ray films (GE Healthcare). BN-PAGE analysis was performed as previously described (43). Isolated mitochondria were solubilized for 10 min in digitonin buffer (1% digitonin, 150 mM NaCl, 50 mM Tris-HCl, pH 7.5, 10% glycerol, 2 mM PMSF), and extracts were clarified by centrifugation for 15 min at 4°C and 20,000 × *g*. Loading dye (10×; 5% Coomassie G-250, 500 mM 6-aminohexanoic acid, 0.1 M bis-Tris, pH 7.0) was added to the supernatant, and samples were separated on a 4 to 13% polyacrylamide gel.

### SILAC labeling and mass spectrometry.

For stable isotope labeling by amino acids in cell culture (SILAC) analysis of Cox20-containing complexes, the *ARG4* gene was deleted from wild-type cells (44) and cells expressing TEV-protein A-7His-tagged Cox20 (Cox20^ProtA^) (see Table S1 in the supplemental material). Yeast cells were cultured on minimal medium (0.67% yeast nitrogen base, appropriate amino acids, 2% galactose) containing either stable isotope-labeled heavy (H) l-arginine (U-^13^C_6_, 99%; U-^15^N_4_, 99%) and l-lysine (U-^13^C_6_, 99%; U-^15^N_2_, 99%) (Cambridge Isotope Laboratories) or the nonlabeled light (L) counterparts. Two independent replicates, including a label switch, were performed. Differentially labeled mitochondria (from wild-type cells and cells expressing Cox20^ProtA^) were isolated, equally pooled, and solubilized. Cox20^ProtA^ complex purification using IgG beads was performed as described above. Eluates were analyzed both by SDS-PAGE and by 4 to 13% gradient BN-PAGE. Gel lanes were cut into 10 slices (SDS gels) or 13 slices (BN gels) of equal sizes. The gel slices were processed for tryptic in-gel digestion, including reduction of disulfide bonds and alkylation of free thiol groups, as described previously (45).

Tryptic peptides were analyzed by liquid chromatography (LC)-MS on an LTQ-Orbitrap XL mass spectrometer (Thermo Scientific, Bremen, Germany) connected to an Ultimate 3000 RSLCnano system (Thermo Scientific, Dreieich, Germany). Peptides were washed and preconcentrated on PepMap C_18_ μ-precolumns (5 mm by 0.3 mm; Thermo Scientific) and separated using a C_18_ reversed-phase nano-LC column (50 cm by 75 μm; particle and pore size, 2 μm and 100 Å, respectively; Acclaim PepMap RSLC column; Thermo Scientific). For peptide elution, a linear gradient of a 35-min duration (samples from SDS-PAGE) or a 55-min duration (samples from BN-PAGE) ranging from 0.3% to 19.5% acetonitrile and 0.5% to 32.5% methanol in 4% dimethyl sulfoxide and 0.1% formic acid was applied. The flow rate was 250 nl/min. The mass spectrometer was operated with settings described before (46).

MS/MS data were processed for protein identification and SILAC-based relative quantification using the MaxQuant/Andromeda software tool (version 1.4.1.2) (47, 48). Searches against the Saccharomyces Genome Database (SGD; www.yeastgenome.org; download version, 2 March 2011) and relative quantification were performed as described previously (46). For the analysis of samples from SDS gels, all 10 slices of a replicate were defined as a single experiment in the MaxQuant experimental design template in order to determine the overall protein SILAC ratios for each replicate. Ratios are reported as Cox20^ProtA^-expressing strain/wild-type ratios (i.e., L/H for replicate 1 and H/L for replicate 2) (see Table S1 in the supplemental material). For the analysis of samples derived from BN gels, each slice was defined as an individual experiment in the experimental design template in order to retrieve information about proteins for each individual slice. Normalized abundance profiles of selected proteins were established on the basis of the sum of all peptide MS intensities assigned to a given protein for each slice and normalized to the highest intensity determined across all slices of a replicate. Depending on the SILAC labeling of the Cox20^ProtA^-expressing cells, the light (replicate 1) or heavy (replicate 2) MS intensity was used (see Table S2).

## RESULTS

### Cox20 participates in two distinct complexes.

**FIG 1:**
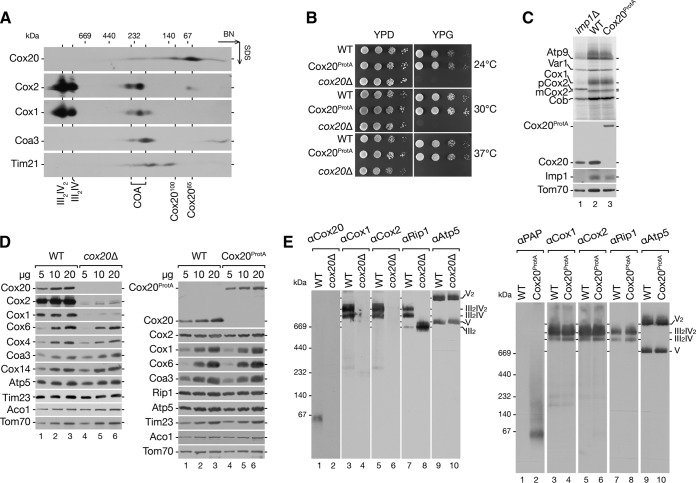
Functionality of a C-terminally tagged Cox20. (A) Endogenous Cox20 is present in two complexes of 65 and 100 kDa. Mitochondria (100 μg) isolated from wild-type cells were solubilized and analyzed by 4 to 13% BN-PAGE followed by two-dimensional SDS-PAGE and Western blotting. (B) Test of Cox20^ProtA^-expressing cell growth. Wild-type (WT) and Cox20^ProtA^-expressing cells were spotted on fermentable (glucose, YPD) and nonfermentable (glycerol, YPG) carbon sources in serial 10-fold dilutions and incubated at the indicated temperatures. The respiration-deficient *cox20*Δ strain was used as a control. (C) Mitochondrial protein synthesis in a Cox20^ProtA^-expressing strain. Mitochondrial translation products of wild-type and Cox20^ProtA^-expressing strains were labeled *in organello* for 20 min. Samples were analyzed by SDS-PAGE and digital autoradiography or Western blotting. As a control, the *imp1*Δ Cox2 processing-deficient strain was used. (D and E) Protein steady-state levels in *cox20*Δ and Cox20^ProtA^-expressing strains. Solubilized mitochondria from wild-type, *cox20*Δ mutant, and Cox20^ProtA^-expressing strains were separated by SDS-PAGE (D) or 4 to 13% BN-PAGE (E) and analyzed by Western blotting using specific antibodies, as indicated.

Cytochrome *c* oxidase assembly occurs through different assembly intermediates, during which Cox1, Cox2, and Cox3 are added to the maturating enzyme in a sequential manner. While Cox1 maturation has been assessed in great detail, the assembly pathway of Cox2 is ill defined. Among Cox2-specific assembly factors, the inner membrane protein Cox20 is critical for its maturation (3, 12, 29, 49). To assess the role of Cox20 in the biogenesis pathway of Cox2, we analyzed the protein composition of Cox20-containing complexes. Therefore, wild-type mitochondria were solubilized in digitonin-containing buffer and analyzed by two-dimensional electrophoresis (BN-PAGE followed by SDS-PAGE) and Western blotting. Cox20 migrated in two distinct complexes at 100 kDa and at 65 kDa, which we termed Cox20^100^ and Cox20^65^, respectively (Fig. 1A). These complexes did not comigrate with respiratory chain supercomplexes (III_2_IV_2_ and III_2_IV) or with the early assembly intermediates of Cox1 (COA complexes) (41, 50, 51).

This finding prompted us to investigate the Cox20 function and interaction network in more detail. To this end, we integrated a protein A tag-encoding cassette into the *COX20* chromosomal locus, allowing expression of a fusion protein under the control of the endogenous *COX20* promoter. The C-terminal protein A tag could be cleaved from the Cox20 portion by tobacco etch virus (TEV) protease treatment. To assess the functionality of the construct, we compared the growth behavior in comparison to that of the wild type and the *cox20*Δ mutant on fermentable (YPD) and nonfermentable (YPG) carbon sources at different temperatures (Fig. 1B). While the *cox20*Δ strain displayed the expected growth defect (12, 29, 52), Cox20^ProtA^-expressing cells exhibited wild-type-like growth, indicating that the fusion protein was functional (Fig. 1B). In addition, considering that Cox20 is required for processing of the Cox2 precursor (pCox2) (29), we analyzed if Cox20^ProtA^ allowed the proper processing of Cox2. Therefore, we labeled mitochondrial translation products in mitochondria isolated from wild-type, *imp1*Δ mutant, and Cox20^ProtA^-expressing cells. Mitochondria from the *imp1*Δ mutant failed to process newly synthesized Cox2 and concomitantly accumulate pCox2 (27, 28, 53) (Fig. 1C, lane 1). Cox2 expression and processing were indistinguishable between mitochondria from wild-type and Cox20^ProtA^-expressing cells, again supporting the functionality of the fusion construct (Fig. 1C, lane 2 versus lane 3). When we compared the steady-state levels of selected mitochondrial proteins from wild-type, *cox20*Δ mutant, and Cox20^ProtA^-expressing cells by Western blotting, mitochondria from the *cox20*Δ mutant showed the expected reduction in the amount of Cox1 and Cox2 (Fig. 1D, left). In contrast, Cox20^ProtA^-containing mitochondria displayed wild-type-like protein levels (Fig. 1D, right). Lastly, we solubilized mitochondria from wild-type, *cox20*Δ mutant, and Cox20^ProtA^-expressing cells and investigated mitochondrial complexes by blue native (BN)-PAGE and Western blot analyses (Fig. 1E). In agreement with previously published findings, mitochondria from *cox20*Δ mutant cells displayed a specific cytochrome *c* oxidase deficiency (Fig. 1E, left). In mitochondria from Cox20^ProtA^-expressing cells, all tested mitochondrial protein complexes were similar to those in the mitochondria from wild-type cells (Fig. 1E, right). Moreover, we were able to detect Cox20^ProtA^-containing complexes in the expected size range for Cox20^100^ and Cox20^65^. In conclusion, Cox20 and Cox20^ProtA^ can be detected in two distinct protein complexes. A Cox20^ProtA^ fusion protein is functional and can be used for further analyses of Cox20-containing complexes.

### The mitochondrial ribosome-associated factor Mba1 forms a complex with Cox20.

**FIG 2:**
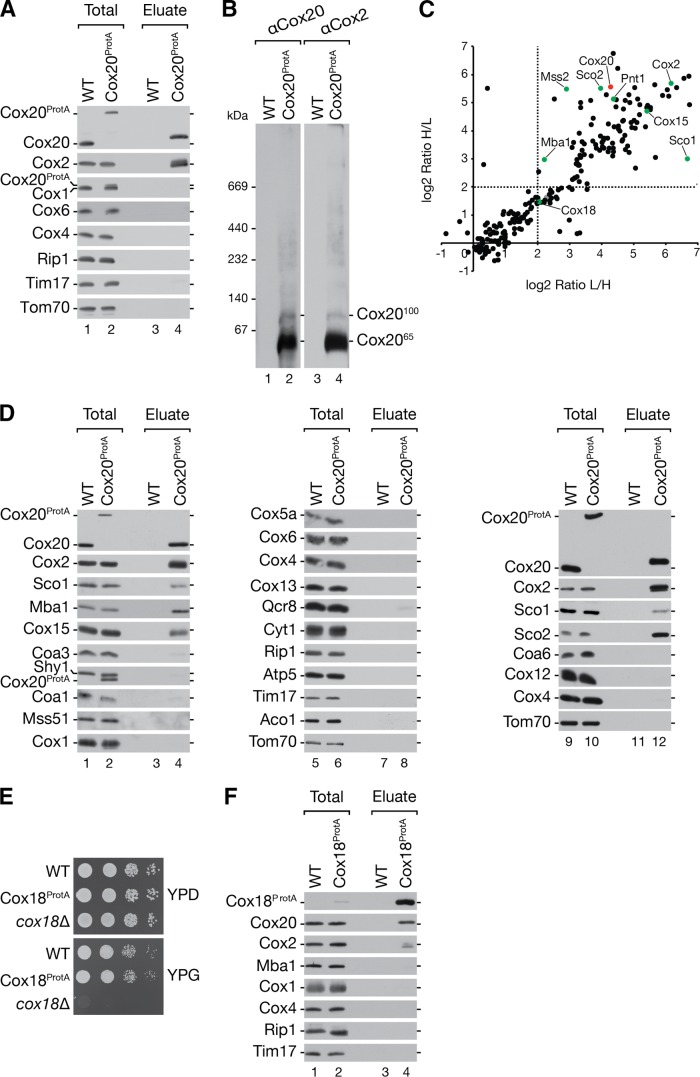
Characterization of Cox20-containing complexes. (A) Cox20 interacts with Cox2. Mitochondria from Cox20^ProtA^ (TEV-protein A-7His-tagged Cox20)-expressing cells were solubilized in digitonin buffer, and the Cox20^ProtA^-containing complexes were natively purified by IgG chromatography and subsequent TEV cleavage. Samples were subjected to SDS-PAGE and Western blotting. Total, 1%; eluate, 100%. (B) Purified Cox20 complexes contain Cox2. Native eluted proteins, as described in the legend to panel A, were analyzed by 4 to 13% BN-PAGE and Western blotting using specific antibodies against Cox20 and Cox2. (C) Mass spectrometric analysis of Cox20^ProtA^ complexes isolated after SILAC. Equal amounts of differentially labeled mitochondria from wild-type (WT) and Cox20^ProtA^-expressing cells were pooled, solubilized, and subjected to IgG chromatography, followed by native elution via TEV cleavage. Eluates were analyzed by SDS-PAGE and LC-MS. Proteins enriched in Cox20^ProtA^ purifications compared to their levels in the wild type with a log_2_ ratio (light/heavy and heavy/light) of >2 were considered potential candidates. Red, Cox20; green, proteins confirmed by Western blotting. (D) Verification of putative Cox20 interaction partners. Mitochondria isolated from Cox20^ProtA^-expressing and wild-type strains were solubilized and subjected to IgG chromatography. Samples were analyzed by SDS-PAGE and Western blotting. (E) Yeast cells from the indicated strains were spotted on medium containing glucose (YPD) or glycerol (YPG) and incubated at 30°C. (F) Mitochondria isolated from wild-type and Cox18^ProtA^-expressing cells were solubilized in digitonin-containing buffer. Protein complexes were isolated via IgG chromatography. Eluates were analyzed by SDS-PAGE and Western blotting.

To define the composition of Cox20-containing complexes, we solubilized Cox20^ProtA^-containing and wild-type mitochondria and purified complexes by IgG chromatography. Bound Cox20-containing protein complexes were released by TEV protease treatment and analyzed by Western blotting. Among the tested cytochrome *c* oxidase subunits, only Cox2 coisolated with Cox20, while control proteins, such as Rip1, Tim17, and Tom70, were not detected in the eluate (Fig. 2A). To analyze intact Cox20-containing complexes, purified fractions were divided, separated by BN-PAGE, and analyzed for the presence of Cox2 and Cox20 by Western blotting (Fig. 2B). Cox20 was detected in the previously described Cox20^65^ and Cox20^100^ complexes, as expected. In addition, Cox2 was present in both complexes (Fig. 2B).

**FIG 3:**
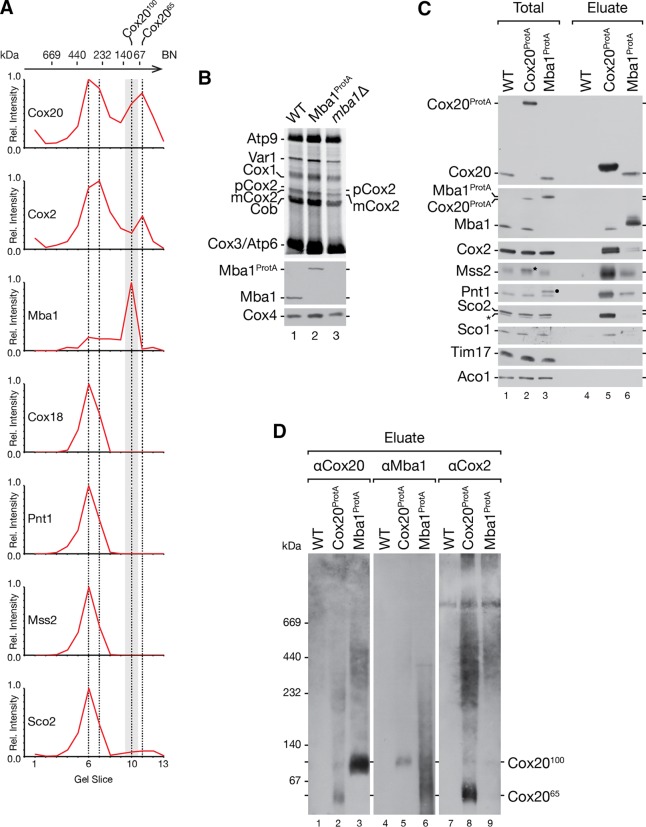
Mba1 and Cox20 form a distinct complex at about 100 kDa. (A) SILAC analysis of differentially labeled Cox20^ProtA^-expressing and control cells. Equal amounts of mitochondria were solubilized and subjected to IgG chromatography, followed by native elution. Eluates were analyzed by BN-PAGE. Gel lanes were cut into 13 slices (slice 1 is at the top, and slice 13 is at the bottom), followed by LC-MS analysis. The normalized intensity profiles of selected proteins copurified with Cox20^ProtA^ are shown. The experiment was repeated with a label swap (see Fig. S1 in the supplemental material). Dashed lines, gel fractions corresponding to the highest-intensity Cox20 peaks; gray box, Cox20^100^ complex; Rel. Intensity, relative intensity. (B) *In organello* labeling was performed for 20 min in mitochondria from wild-type (WT), Mba1^ProtA^-expressing, and *mba1*Δ mutant cells. Mitochondrial translation products were analyzed by SDS-PAGE and Western blotting or digital autoradiography. (C) Cox20 interacts with Mba1. Protein complexes were isolated from mitochondria from cells expressing Cox20^ProtA^ and Mba1^ProtA^ (TEV-protein A-7His-tagged Mba1) by IgG affinity chromatography and analyzed by SDS-PAGE and Western blotting. Asterisk, a cross-reactive signal; star, Cox20^ProtA^ signal; dot, Mba1^ProtA^ signal. Total, 1%; eluate, 100%. (D) Mba1 associates with the Cox20^100^ complex. Eluate from the assay whose results are presented in panel C was analyzed by 4 to 13% BN-PAGE for Cox20^ProtA^- and Mba1^ProtA^-containing complexes.

For a global assessment of the Cox20 interaction network, cells were metabolically labeled using stable isotope labeling by amino acids in cell culture (SILAC) analysis (54) (see Table S1 in the supplemental material). For this, Cox20^ProtA^-containing or control cells were cultured in either heavy or light amino acid-containing medium. In addition to Cox20 and Cox2, mass spectrometric analyses demonstrated an enrichment of proteins implicated in Cox2 biogenesis and the heme A synthase, Cox15 (55–57) (Fig. 2C). Unexpectedly, the ribosome receptor Mba1 was also identified within the Cox20 interaction network (Fig. 2C). Mba1 has been implicated in the process of insertion of newly synthesized translation products by aligning the mitochondrial ribosome with the membrane insertion machinery (22–25). To support the mass spectrometric data, Cox20^ProtA^-containing complexes were purified from mitochondria, and the purified fraction was analyzed by Western blotting (Fig. 2D and 3C, lane 5). In addition, Mba1 and Cox15 were confirmed to copurify with Cox20 (Fig. 2D, lane 4). Interestingly, the other metallochaperone, Coa6, was not detected (Fig. 2D, lane 12). Other inner membrane space twin CX_9_C motif-containing proteins (e.g., Cmc1, Cox19, Cox23) were not identified in the mass spectrometric analysis. As a control we assessed the presence of Cox1 assembly factors. COA complex components (e.g., Coa1, Shy1, and Mss51) could not be detected in the eluate. Structural subunits of the cytochrome *c* oxidase (e.g., Cox4, Cox5a, Cox6, Cox12, and Cox13) and subunits of the cytochrome *bc*_1_ complex (e.g., Cyt1, Rip1, and Qcr8) were also not present in the eluate (Fig. 2D).

Despite an enrichment of Mss2 and Pnt1, proteins implicated in the Cox2 C-terminal translocation machinery, the putative C-terminal Cox2 translocase protein Cox18 was identified in the sample but was less enriched. Due to the lack of a Cox18 antibody, we generated a strain expressing TEV-protein A-7His-tagged Cox18 (Cox18^ProtA^) to investigate the association of Cox18 with Cox20. To assess the functionality of the tagged protein, growth analysis of the Cox18^ProtA^-expressing strain was performed (Fig. 2E). Strains were analyzed for growth on fermentable and nonfermentable carbon sources. In contrast to the respiration-deficient *cox18*Δ strain, the Cox18^ProtA^-expressing strain grew well under all the conditions tested, indicating that tagged Cox18 is functional. We then isolated Cox18^ProtA^ complexes and analyzed them by Western blotting (Fig. 2F). Cox20 and Cox2 could be identified in the eluate, in agreement with the results of previous analyses (12). Interestingly, Mba1 was not recovered in the eluate of the Cox18^ProtA^ isolation (Fig. 2F). These findings underline the presence of Cox20 in several distinct protein complexes. Furthermore, the association of the Cox2 C-terminal export machinery and Mba1 with Cox20 implicates a close functional connection between the first steps of Cox2 biogenesis, N-terminal maturation, the mitochondrial ribosome, and the C-terminal export machinery.

### Mba1 is a constituent of the Cox20^100^ complex.

To determine the protein composition of the Cox20^65^ and Cox20^100^ complexes, we carried out quantitative mass spectrometric analysis of these protein complexes after separation by BN-PAGE. Therefore, we cultured wild-type and Cox20^ProtA^-containing cells in SILAC medium and isolated native complexes. The eluates were loaded onto a single BN-polyacrylamide gel lane, which was cut into 13 slices for mass spectrometric analyses (see Table S2 in the supplemental material). The normalized abundance profiles of selected proteins coisolated with Cox20^ProtA^ were plotted against the gel slices (Fig. 3A; see also Fig. S1). The abundance distribution of Cox20 along the BN-polyacrylamide gel lane displayed two main peaks in slices 6 and 7 (approximately 400 to 250 kDa) and slices 10 and 11 (approximately 140 to 60 kDa). The Cox2 profile matched the Cox20 profile. Slices 6 and 7 contained proteins involved in the Cox2 C-terminal export machinery (Mss2, Pnt1, and Cox18) and Sco2, which participates in copper insertion into Cox2. Slices 10 and 11 included the Cox20^65^ and the Cox20^100^ complexes. Interestingly, Mba1 displayed a maximal abundance in slice 10, while Cox20 and Cox2 peaked in slice 11 (Fig. 3A).

To support the finding that Mba1 was selectively present in Cox20^100^, *MBA1* was chromosomally tagged to encode a fusion protein with a C-terminal protein A extension. Using *in organello* labeling, we confirmed the functionality of tagged Mba1 (Fig. 3B). Indeed, TEV-protein A-7His-tagged Mba1 (Mba1^ProtA^) exhibited a wild-type-like Cox2 processing efficiency, in contrast to its respective deletion strain, in which there was an accumulation of pCox2. Subsequently, Mba1-containing complexes were purified by IgG chromatography and analyzed by Western blotting (Fig. 3C). In comparison to the findings with Cox20 purification, smaller amounts of Pnt1, Mss2, and Cox2 coisolated with Mba1. However, the copper chaperones Sco1 and Sco2 did not copurify with Mba1. These results support the idea that Mba1 associates with Cox2 assembly but not the copper insertion machinery. The low level but reproducible coisolation of Cox2 with Mba1 suggests that only a small population of Cox20 complexes contains Mba1. To directly assess the amount of Mba1 in Cox20^100^, we isolated Cox20^ProtA^- and Mba1^ProtA^-containing complexes under native conditions and subjected the eluate to BN-PAGE and Western blotting (Fig. 3D). Membrane probing for Cox20 or Mba1 identified Cox20^100^ as the Mba1-containing complex (Fig. 3D, lanes 1 to 6), while the majority of Cox2 was identified in Cox20^65^ (Fig. 3D, lanes 7 to 9). The Cox20 antibody is directed against the C terminus of Cox20. After cleavage of the protein A portion from Cox20^ProtA^, the remaining spacer amino acids affected recognition by the Cox20 antibody. We conclude that Mba1 and Cox20 form the 100-kDa complex, while Cox20^65^ comprises Cox2 and Cox20 but lacks Mba1.

### Mba1 and Cox20 interact with the mitochondrial ribosome to initiate Cox2 assembly.

**FIG 4:**
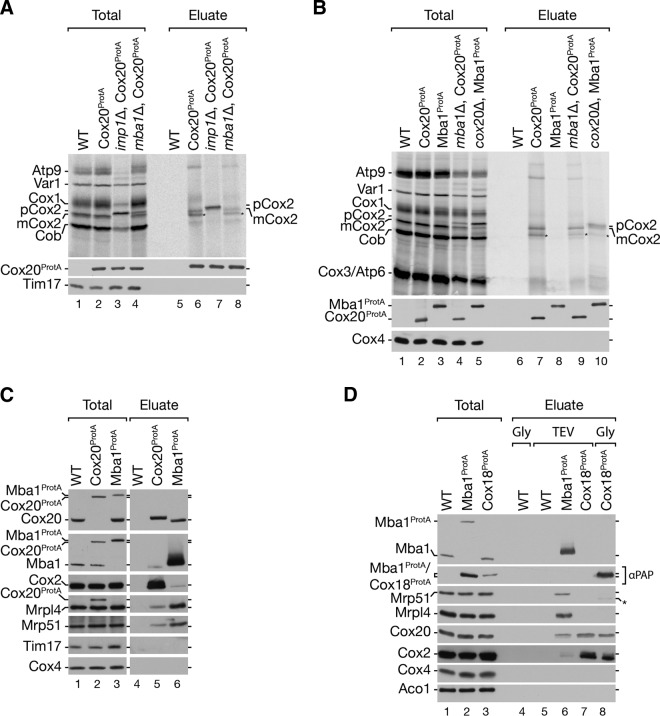
Role of Mba1 in Cox2 synthesis and assembly. (A) Mba1 is required for the association of Cox2 assembly factors with unprocessed Cox2. Mitochondria were isolated from the indicated strains, and mitochondrial translation products were radiolabeled *in organello* for 20 min. After solubilization of the mitochondria with digitonin, IgG affinity chromatography was performed and samples were analyzed by SDS-PAGE and digital autoradiography or Western blotting. Total, 10%; eluate, 100%. (B) Mba1 binds newly synthesized unprocessed and matured Cox2 in the absence of Cox20. ProtA isolation was performed from the indicated strains after *in organello* labeling of mitochondrial translation products. Samples were separated by SDS-PAGE and analyzed by digital autoradiography or Western blotting. Asterisks, a Cox2 isoform; pCox2, Cox2 precursor; mCox2, mature Cox2. Total, 10%; eluate, 100%. (C) Cox20 associates with Mba1 and the mitochondrial ribosome. Western blotting was used to analyze the samples for Cox20^ProtA^ and Mba1^ProtA^. The indicated antibodies against components of the mitochondrial ribosomal large subunit (Mrpl4) and small subunit (Mrp51) were used. Total, 1%; eluate, 100%. (D) Mba1 does not associate with late Cox2 assembly factor Cox18. IgG affinity purification was used to isolate Mba1^ProtA^ and Cox18^ProtA^ from mitochondria. Bound proteins were eluted by TEV cleavage (TEV) or glycine, pH 2.8 (Gly). The Mba1^ProtA^ and the Cox18^ProtA^ signals were detected using anti-ProtA (αPAP) antibody. Asterisk, a cross-reaction. Total, 1%; eluate, 100%.

Previous studies defined Mba1 as a mitochondrial ribosome receptor required for respiratory chain biogenesis (20, 22, 24). The identification of Cox20^100^ as a Cox20-Mba1-specific complex prompted us to investigate its function in Cox2 translation and maturation. To this end, we deleted *IMP1* and *MBA1* from a Cox20^ProtA^-expressing yeast strain and radioactively labeled mitochondrial translation products prior to purification via IgG chromatography (Fig. 4A). Cox20^ProtA^ copurified with mature Cox2 (mCox2) in the wild-type background and exclusively with pCox2 in the *imp1*Δ strain (Fig. 4A, lanes 6 and 7). Interestingly, despite the accumulation of pCox2 in mitochondria from *mba1*Δ and *imp1*Δ strains (Fig. 4A, lanes 3 and 4), pCox2 was not recovered from *mba1*Δ strain mitochondria by Cox20^ProtA^ (Fig. 4A, lane 8). We speculated that Mba1 is required for the efficient transfer of pCox2 to Cox20. To support this hypothesis, we performed a complementary analysis in which we investigated the Mba1-Cox2 association in the absence of Cox20 (Fig. 4B). Therefore, we generated an Mba1^ProtA^-expressing strain and an Mba1^ProtA^-expressing *cox20*Δ strain and compared the association of newly synthetized Cox2. For comparison, we purified Cox20^ProtA^ from *mba1*Δ cells (see above). In agreement with the findings of Preuss et al. (24), no mitochondrial translation products copurified with Mba1^ProtA^ in the absence of a chemical cross-linker (Fig. 4B, lane 8). However, in the absence of Cox20, Mba1 was coisolated with mature Cox2 as well as the precursor, pCox2 (Fig. 4B, lane 10). Accordingly, when Cox20 was missing, Cox2 accumulated in an Mba1-containing complex. Despite the absence of Cox20, Cox2 underwent proteolytic maturation to some extent. These findings are in agreement with Mba1 acting upstream of Cox20 in the biogenesis of Cox2.

The observed complex between Cox20 and Mba1 suggested that Cox20 could be in close proximity to the mitochondrial ribosome. To test this directly, we purified Cox20^ProtA^ and Mba1^ProtA^ from mitochondria. Both Cox20^ProtA^ and Mba1^ProtA^ coisolated with Mrpl4, a member of the large ribosomal subunits, and Mrp51, a component of the small ribosomal subunit (Fig. 4C). Hence, both Cox20 and Mba1 associate with mitochondrial ribosomes. To investigate the ribosome association along the Cox2 assembly line, we analyzed if Cox18, acting at a later stage in Cox2 maturation, also displayed a ribosome association. Mba1^ProtA^- and Cox18^ProtA^-containing complexes were purified and eluted from the column by TEV protease cleavage. In addition, due to the lack of a Cox18 antibody, in a second identical Cox18^ProtA^ isolation, Cox18^ProtA^ was eluted by use of a pH shift to release the tagged protein from the IgG resin. Cox20 was detected in Cox18 and Mba1 isolations (Fig. 4D, lanes 6 to 8). In contrast, ribosomal proteins were identified only in the Mba1^ProtA^ isolation. Accordingly, an interaction of Mba1 and Cox20 with the ribosome is restricted to the early steps of Cox2 maturation but is apparently lost when Cox18 engages Cox20 and Cox2 (Fig. 4D, lane 6).

### Translation of Cox2 is a prerequisite for Mba1-Cox20 interaction with ribosomes.

**FIG 5:**
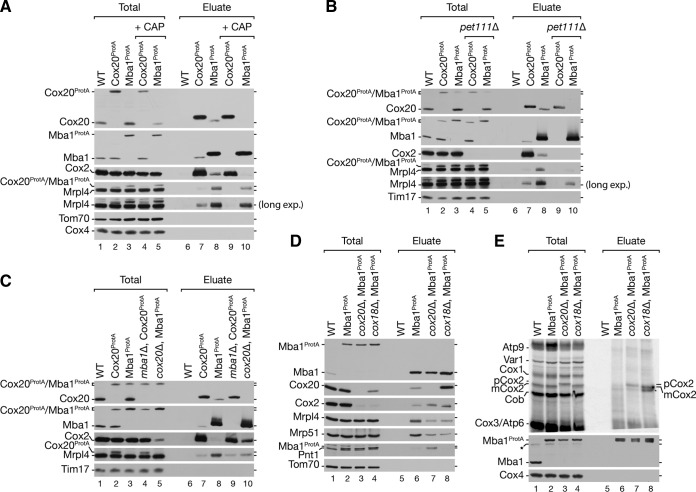
Defects in processing of Cox2 sequester Mba1 with unassembled Cox2. (A and B) Cox20 and Mba1 interact in a Cox2-dependent manner. Mitochondria were isolated from wild-type and *pet111*Δ mutant strains expressing Cox20^ProtA^ or Mba1^ProtA^. Cells were pretreated with 6 mM chloramphenicol (CAP) for 3 h, where indicated. Solubilized mitochondria were subjected to IgG chromatography and analyzed by SDS-PAGE and Western blotting. Total, 1%; eluate, 100%. long exp., long exposure. (C) The Cox20 ribosome association is Mba1 independent. Protein complexes were purified from the indicated deletion mutants expressing Cox20^ProtA^ and Mba1^ProtA^ by IgG affinity chromatography. Total, 1%; eluate, 100%. (D) Cox2 assembly defects lead to a reduce Mba1 association with the ribosome. Mba1^ProtA^ was isolated from the indicated strains. Samples were analyzed by SDS-PAGE and Western blotting. Total, 1%; eluate, 100%. (E) Mitochondria were isolated from the indicated strains, and *in organello* labeling of mitochondrial translation products was performed for 20 min. Subsequently, the mitochondria were solubilized, subjected to IgG chromatography, and analyzed by SDS-PAGE and Western blot analysis or digital autoradiography. Total, 10%; eluate, 100%. Black star, antibody cross-reaction; asterisk, aberrantly migrating form of mature Cox2.

We hypothesized that actively translating ribosomes are required for the formation of the Mba1-Cox20 complex. To test this, we treated yeast cells with the translational inhibitor chloramphenicol (CAP) and isolated Cox20^ProtA^- and Mba1^ProtA^-containing protein complexes under native conditions (Fig. 5A). In the presence of chloramphenicol, the Cox20-Mba1 interaction was lost. In addition, ribosomes were no longer purified by Cox20 (Fig. 5A, lane 7 versus lane 9). In contrast, chloramphenicol had little impact on the ribosome interaction with Mba1 (Fig. 5A, lane 8 versus lane 10). We concluded that mitochondrial translation is required for formation of the Mba1-Cox20 complex with the ribosome.

Since CAP is a general mitochondrial protein translation inhibitor, we generated *pet111*Δ strains with protein A-tagged Cox20 and Mba1. Pet111 is a translational activator for Cox2 mRNA. Deletion of *PET111* abolishes Cox2 synthesis (13, 14, 58). While Cox20^ProtA^ and Mba1^ProtA^ isolated Cox2 and ribosomes in wild-type mitochondria, Mba1^ProtA^ isolated significantly less ribosome from *pet111*Δ mitochondria (Fig. 5B, lane 8 versus lane 10). Moreover, in the absence of Pet111, Cox20^ProtA^ did not purify ribosomes anymore (Fig. 5B, lane 7 versus lane 9). More interestingly, the loss of Cox2 translation also abolished the interaction between Cox20 and Mba1 (Fig. 5B). Accordingly, the Mba1-Cox20 interaction depends on Cox2 translation. This finding is in agreement with the observation that a loss of Mba1 did not disturb the interaction of newly synthesized mCox2 with Cox20 but that depletion of Cox20 leads to an accumulation of pCox2 with Mba1 (Fig. 4A, lanes 9 and 10).

We next investigated the interaction of Cox20 and Mba1 with mitochondrial ribosomes in mitochondria from *mba1*Δ and *cox20*Δ strains. As seen before, Cox2 accumulated with Mba1 in mitochondria from the *cox20*Δ strain (Fig. 5C, lanes 5 and 10). Surprisingly, the interaction of mitochondrial ribosomes with Mba1 was reduced when Cox20 was lacking (Fig. 5C, lane 10). This finding suggests that during Cox2 maturation Mba1 interacts with Cox2 independently of the ribosome. We hypothesized that a disruption of Cox2 biogenesis at a later stage would lead to an accumulation of Cox2 and Cox20 with Mba1, while at the same time the ribosome would be dissociated. Hence, we isolated Mba1^ProtA^-containing complexes from a *cox18*Δ strain background (Fig. 5D). As predicted, significantly more Cox20 and Cox2 were isolated with Mba1, while substantially fewer ribosomes were associated (Fig. 5D, lane 8). These data suggest that the Cox20-Mba1 complex acts upstream of the Cox2–C-terminal tail translocase Cox18.

Previous work by Saracco and Fox (2002) (34) reported that deletion of Cox18 leads to an accumulation of processed unassembled Cox2 (mCox2). To determine if the interaction of Mba1 with the mature Cox2 nascent chain is altered in the *cox18*Δ strain, we radiolabeled mitochondrial translation products and isolated Mba1^ProtA^-containing complexes. As expected, an accumulation of processed Cox2 (mCox2) with Mba1 was apparent in the absence of Cox18 (Fig. 5E, lanes 6 and 8).

In summary, we found that Mba1 dynamically associates with Cox20 and early Cox2 assemblies at different stages of the biogenesis pathway. We conclude that Mba1 promotes Cox2 assembly by mediating the shuttling of newly synthesized Cox2 from the insertion machinery to the C-terminal export machinery in a Cox20-dependent manner.

## DISCUSSION

Cox20 is a conserved protein that was previously defined to be a Cox2-specific chaperone. This classification is based on the observation that the majority of newly synthesized Cox2 associates with Cox20 in mutant mitochondria affected in Cox2 incorporation into assembly intermediates (29, 59). Here, we defined the following to be Cox20-containing complexes: a trail of complexes at 200 kDa, the Cox20^100^ complex, and the Cox20^65^ complex. In agreement with the suggested Cox2 chaperone function of Cox20, all detected Cox20 assemblies contained Cox2, but they did not contain any other structural subunit of cytochrome *c* oxidase. Additional proteins implicated in Cox2 C-terminal processing, copper insertion, and protein export were identified in the Cox20 complexes at 200 kDa and in the Cox20^100^ complex. The Cox2 C-terminal export factor Cox18, together with Mss2 and Pnt1, were found in the trail of complexes at 200 kDa. This finding confirms the previously described interactions and suggests that these proteins act as a C-terminal export complex (34, 60, 61). However, the association of Cox20 with Mss2, Pnt1, and Sco2 has not been reported before. In mammals, the homolog of Cox20 was found in complex with the copper chaperones SCO1 and SCO2 (both represent homologs of yeast Sco1) (30). However, the molecular function of Cox20 in the copper insertion process remains elusive. In summary, these observations implicate Cox20 in several different processes during Cox2 biogenesis and agree with a role of Cox20 as a scaffold protein.

**FIG 6:**
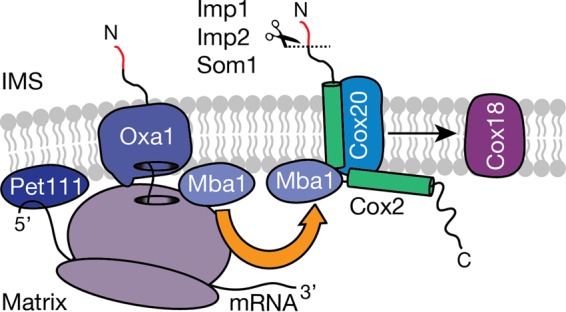
Ribosome-associated Mba1 forms a specific complex with Cox20. *COX2* mRNA is translated with the help of the membrane-bound translational activator Pet111. Subsequently, the N-terminal tail is cotranslationally exported into the intermembrane space (IMS) via Oxa1. Mba1 shuttles newly synthesized Cox2 from the export machinery (Oxa1) to the Cox20 protein, promoting its maturation, which requires downstream assembly intermediates like the Cox2 C-terminal tail translocase, Cox18. Mba1 therefore links the cotranslational export of the N-terminal tail of Cox2 with its C-tail translocation.

The translocation of the Cox2 C terminus is considered to occur posttranslationally (62). Indeed, an association between Cox18 and the ribosome or Cox18 and the mitochondrial ribosome-associated Mba1 has not been detected. The identification of Mba1 in the Cox20^100^ complex was an unexpected finding. Previous publications implicated Mba1 in the cotranslational export of mitochondrion-encoded proteins of complexes III and IV and stabilization of Cox2 (20, 22, 24). However, a mechanism by which Mba1 assists with Cox2 maturation has not yet been defined. Here we found an Mba1 interaction with Cox2 assembly intermediates (Fig. 6). In contrast to Cox20, we did not detect interactions between Mba1 and the copper chaperones Sco1 and Sco2, supporting the hypothesis that different pools of Cox20 exist. In addition, this finding indicates that the Cox20-Mba1 interaction is upstream of copper insertion into Cox2. The cooperation of Mba1 with Cox20 in the maturation of Cox2 can be suggested on the basis of the finding of a Cox20-Mba1 complex. In the absence of Cox20 and Cox18, Mba1 isolates with newly synthesized Cox2, suggesting that Mba1 escorts Cox2 during assembly. However, at steady state an association of Mba1 with newly synthesized Cox2 could not be identified. It appears likely that the Mba1-Cox2 interaction is transient but can be stabilized only in the absence of downstream assembly factors, such as Cox20 or Cox18. In agreement with this hypothesis, Mba1 is required for the interaction of pCox2 with Cox20. In contrast, the association of Cox20 with mature Cox2 is Mba1 independent. This is supported by the weak phenotype of an *mba1*Δ mutant, which displayed only a weak Cox2 assembly defect, indicative of Mba1-independent pathways (22, 24).

Recent cryo-electron tomography studies localized Mba1 in proximity to the ribosomal exit tunnel (22, 23, 63). However, unlike its human counterpart, MRPL45, Mba1 is not a structural component of the ribosome. Accordingly, Mba1 might be dynamically associated with the ribosome. In support of this, we found that Cox2 assembly defects affected the association of Mba1 with the ribosome. A similar phenotype could also be observed in the *pet111*Δ mutant, in which the Mba1 interaction with the ribosome was decreased but not abolished, indicating that Mba1 preferentially binds to ribosomes translating Cox2 (13, 14). Our analyses unexpectedly revealed an association between Cox20 and the translating mitochondrial ribosome. This interaction was dependent on Cox2 translation. Since Cox20 lacks a significant matrix-exposed domain, the Cox20-ribosome interaction is unlikely to be direct. We speculate that a third factor could mediate this association. The nascent Cox2 would in fact be an attractive candidate for this.

The formation of the Cox20-Mba1 complex appears to be dynamic. Since the Cox20-Mba1 interaction requires Cox2, we suggest that Cox2 links the association between these proteins. Notably, in the Cox20-null mutant, Mba1 is sequestered with Cox2 and components of the C-terminal translocation complex. Instead, in the absence of Cox18, Mba1 accumulates with Cox20. These findings sustain a dynamic distribution of Mba1 between different Cox2 maturing assemblies. Collectively, the results of these analyses suggest that at the ribosome Mba1 shuttles newly synthesized Cox2 to Cox20 to support Cox2 maturation. Subsequently, Mba1 remains associated with Cox2 to facilitate transfer of the polypeptide to the tail export machinery in the inner membrane.

## Supplementary Material

Supplemental material
