# Supplementary material for: Ribosome-Associated Mba1 Escorts Cox2 from Insertion Machinery to Maturing Assembly Intermediates
Source: Mol Cell Biol. 2016 Oct 28;36(22):2782–93. doi: 10.1128/MCB.00361-16 (PMC5086520; doi:10.1128/MCB.00361-16)
Supplement: Supplemental material [file supp_36_22_2782__index.html]

Supplemental material 

# Ribosome-Associated Mba1 Escorts Cox2 from Insertion Machinery to Maturing Assembly Intermediates

## Supplemental material

- Supplemental file 1 -

  Fig. S1 (MS analysis of Cox20ProtA isolated complexes after SILAC labeling)

  PDF, 589K
- Supplemental file 2 -

  Table S1 (SILAC-based quantitative MS analyses of affinity-purified Cox20 complexes)

  XLSX, 102K
- Supplemental file 3 -

  Table S2 (MS-based establishment of protein abundance profiles of affinity-purified Cox20 complexes separated by BN-PAGE)

  XLSX, 22K
