# Supplementary material for: Ribosome-Associated Mba1 Escorts Cox2 from Insertion Machinery to Maturing Assembly Intermediates
Source: Mol Cell Biol. 2016 Oct 28;36(22):2782–93. doi: 10.1128/MCB.00361-16 (PMC5086520; doi:10.1128/MCB.00361-16)
Supplement: Supplemental material [file MCB.00361-16_zmb999101339so1.pdf]

A

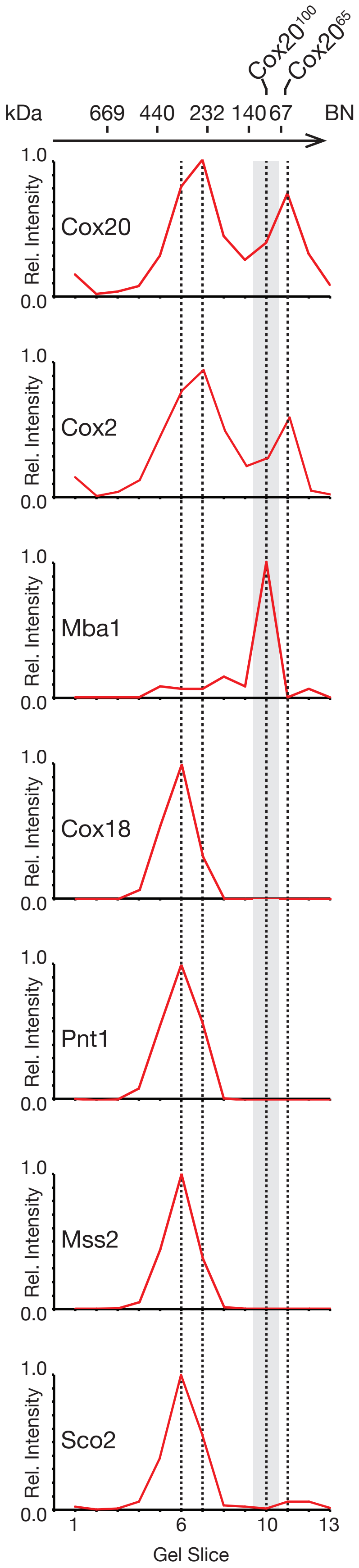

(A) Mass spectrometric analysis of Cox20^{ProtA} isolated complexes after SILAC labeling. Equal amounts of Cox20^{ProtA} light labeled mitochondria were mixed with the respective wild-type heavy labeled mitochondria, solubilized and subjected to IgG chromatography. Eluates were analyzed as described in Figure 3A. Dashed lines: gel fractions corresponding to the highest intensity Cox20 peaks. The Gray box indicates Cox20^{100} complex.
